# Improving screening for antibody deficiency using calculated globulin and serum protein electrophoresis

**DOI:** 10.3389/fimmu.2026.1734197

**Published:** 2026-03-18

**Authors:** Linda Mokrane, Azzeddine Tahiat, Sihem Taguemount, Hassen Messaoudi, Nadia Boukhenfouf, Abdelbassat Ketfi, Leila Smati, Rachida Boukari, Kamel Djenouhat

**Affiliations:** 1 Department of Medical Biology, Rouiba Hospital, Algiers University of Health Sciences, Algiers, Algeria; 2 Department of Internal Medicine, Rouiba Hospital, Algiers University of Health Sciences, Algiers, Algeria; 3 Department of Pediatrics, Rouiba Hospital, Algiers, Algeria; 4 Department of Pneumology, Rouiba Hospital, Algiers University of Health Sciences, Algiers, Algeria; 5 Department of Pediatrics, Bologhine Hospital, Algiers University of Health Sciences, Algiers, Algeria; 6 Department of Pediatrics, Mustapha University Hospital, Algiers University of Health Sciences, Algiers, Algeria

**Keywords:** calculated globulin, serum protein electrophoresis, screening, common variable immunodeficiency, hypogammaglobulinemia, antibody deficiency

## Abstract

**Purpose:**

Antibody deficiencies, particularly Common Variable Immunodeficiency (CVID), represent a major health concern due to comorbidities linked to delayed diagnosis. Calculated globulin (CG) and the gammaglobulins derived from serum protein electrophoresis have been proposed as reliable indices for screening hypogammaglobulinemia. This study aimed to establish optimal cut-offs for CG and the gammaglobulins to identify patients with low IgG among hospitalized adolescents and adults from Algeria.

**Methods:**

Serum samples and clinical data were collected from hospitalized patients aged over 10 years. Total protein and albumin were determined by the biuret and bromocresol green methods, IgG was quantified by nephelometry, and gammaglobulins were determined by gel or capillary serum protein electrophoresis. In addition, a control group was included for cut-off verification.

**Results:**

A total of 980 patients were recruited, including 762 adults (≥ 18 years) and 218 adolescents (10–17 years), with a median age of 47 years. The control group comprised 20 patients with confirmed CVID. The most frequent clinical manifestations were autoimmune disorders (33%), inflammatory disorders (31%), and severe infections (24%). Hypogammaglobulinemia was identified in 94 patients, including 6 with primary causes. IgG levels correlated significantly with both CG and gammaglobulins. Linear regression analysis showed that IgG accounted for 49% of the variance in CG and 75% in the gammaglobulins. A CG cut-off value of 22 g/L yielded a sensitivity of 80% and a specificity of 87%, whereas a gammaglobulin cut-off of 8.9 g/L achieved a sensitivity of 96% and a specificity of 88%. Gammaglobulin cut-off values were consistent between adult and pediatric groups, while differences in CG cut-offs were observed (adult group: 22.25 g/L; pediatric group: 24.15 g/L). Thirteen of the 20 patients with CVID in the control group (65%) had a CG level below 22 g/L.

**Conclusion:**

CG and serum protein electrophoresis are reliable tools for screening hypogammaglobulinemia. Although both tests demonstrated satisfactory sensitivity and specificity, certain cases may be underestimated or overestimated. Therefore, thorough clinical evaluation and, whenever possible, direct immunoglobulin measurement remain essential.

## 1 Introduction

Immunoglobulins (Ig) are the key proteins involved in humoral adaptive immunity, produced by B lymphocytes in response to a wide variety of antigens. Among the five major Ig isotypes (IgG, IgA, IgM, IgD, and IgE) identified to date, IgG is the most abundant, representing nearly 80% of circulating Ig in the human plasma (1). IgG comprises four subclasses, each with distinct structural and functional properties, contributing to broad-spectrum immune protection and essential homeostatic functions (2). A significant decrease in Ig levels, also known as hypogammaglobulinemia, can substantially increase susceptibility to infections and lead to immune dysregulation (3). Hypogammaglobulinemia may result from primary genetic defects or, more commonly, from secondary causes, such as drug-induced immunosuppression, infectious diseases, malignancies (e.g., lymphoma), or excessive protein loss (4, 5).

Primary antibody deficiencies (PADs) are a group of genetic disorders that impair the development and/or maturation of B cells, resulting in reduced production of one or more Ig isotypes (6). Common Variable Immunodeficiency (CVID) is the most prevalent symptomatic PAD (7). It encompasses a heterogeneous group of disorders characterized by hypogammaglobulinemia. The clinical manifestations of CVID range from recurrent infections, primarily affecting the respiratory tract, to atypical non-infectious manifestations such as polyclonal lymphoproliferation, inflammatory disorders, autoimmune diseases, and malignancies (7–9). Despite advances in genetic testing, pathogenic mutations are identified in only 25% to 30% of cases (10), making the diagnosis mainly dependent on clinical and immunological findings. Another unusual feature of CVID is its variable age of onset: unlike most inborn errors of immunity (IEI) that present in infancy, the onset of CVID can occur anytime from early childhood to late adulthood (11). These atypical features often complicate the diagnostic process and contribute to delays in diagnosis (12).

Since the first diagnostic criteria for CVID were established by the European Society for Immunodeficiencies (ESID) and the Pan-American Group for Immunodeficiency (PAGID) in 1999 (4), several updates have been proposed to reduce diagnostic delays. These include the criteria proposed by Ameratunga et al. in 2013 (13), the International Consensus (ICON) criteria in 2015 (14), and the revised ESID registry criteria in 2019 (15). Across these four sets of criteria, key diagnostic features are consistently shared: low IgG in combination with low IgA and/or IgM, poor vaccine responses, and the exclusion of secondary causes of hypogammaglobulinemia. Compared to the original 1999 ESID/PAGID criteria, which did not consider clinical manifestations and set the minimum age of diagnosis at 2 years, the updated criteria have increased the age threshold to 4 years, and have involved a more detailed account of both infectious and non-infectious clinical features, as well as histological findings. Newer criteria have also considered additional immunological assessment, including reduced levels of switched memory B cells (13–15), and analysis of the T cell compartment to help distinguish CVID from combined immunodeficiencies, which may also present with hypogammaglobulinemia (15).

As hypogammaglobulinemia, particularly reduced IgG, is the hallmark of CVID, the diagnostic workup must include measurement of Ig levels. The primary automated laboratory techniques used for this purpose are nephelometry and turbidimetry (16). However, in low-income settings, limited availability and high costs can restrict routine Ig testing, particularly in adults with non-specific symptoms. This highlights the need for accessible and affordable screening tools to support early diagnosis.

To address this issue, Jolles et al. (17) proposed the use of Calculated Globulin (CG) as a cost-effective and practical screening index for detecting hypogammaglobulinemia. CG represents the concentration of serum globulins, calculated by subtracting albumin from total protein (TP). TP is typically measured using the Biuret method, while albumin is measured through multiple techniques such as nephelometry, the Bromocresol Green or the Bromocresol Purple methods (18). Therefore, the threshold for CG in detecting low IgG levels may differ through techniques. Another useful screening method is serum protein electrophoresis (SPE), which, although semi-quantitative, provides a reliable estimation of the gammaglobulin concentration.

Several studies have evaluated the use of CG and SPE as screening tools for hypogammaglobulinemia, particularly in the context of CVID (17, 19–26). However, the proposed cut-off values for CG and gammaglobulin levels have varied across studies, as have the reported sensitivities and specificities of each test. These discrepancies are likely attributable to differences in sample size, disease categories included, ethnic background, and laboratory methodologies. Another challenge is the influence of age, as protein production differs significantly between children and adults. To date, only few studies have assessed the performance of CG specifically in pediatric populations (26, 27), and none have evaluated screening outcomes simultaneously in both adults and children.

This study is the first in both Africa and the Middle East and North Africa (MENA) region to investigate the diagnostic accuracy of CG and SPE for detecting hypogammaglobulinemia in a large cohort of hospitalized adolescents and adults presenting with immune-related disorders. We aimed to determine the optimal cut-off values for CG and gammaglobulin concentrations that provide the highest sensitivity and specificity for identifying patients with low serum IgG levels.

## 2 Methods

### 2.1 Patient enrollment and study design:

The study was conducted at the Medical Biology Laboratory of Rouiba Public Hospital, Algiers, Algeria. It included a large cohort of hospitalized patients (aged over 10 years), recruited between June 2024 and June 2025. Participants were admitted to the pneumology, internal medicine, or pediatric departments, and recruitment focused on individuals diagnosed with infectious diseases, inflammatory disorders, autoimmune diseases, polyclonal lymphoproliferation, allergies, or malignancies. Patients with multiple myeloma were excluded. For each participant, serum samples and relevant clinical data, including diagnosis, symptoms, symptom onset, and treatment, were collected.

In addition, a control group of patients with a confirmed diagnosis of CVID was included to evaluate the diagnostic performance of CG in identifying CVID. The study protocol was approved by the local ethics committee in accordance with the Declaration of Helsinki.

### 2.2 Laboratory assessment:

For each serum sample, CG was determined by subtracting albumin from TP, based on the results of TP and albumin measurements. IgG levels were measured in all samples and served as the gold standard for detecting hypogammaglobulinemia, defined as IgG <6 g/L in adults, and values below the manufacturer’s reference range in pediatric patients (IgG <5.65 g/L for children aged between 10 and 12 years, and IgG <6 g/L for adolescents older than 12 years) (28). TP was measured using the Biuret method, and albumin was measured using the Bromocresol Green method (Roche Diagnostics, USA). Analyses were performed on the Cobas INTEGRA 400 plus analyzer. IgG levels were quantified by laser nephelometry using the BN2 ProSpec (Siemens Healthineers, Germany). Daily controls were run for quality assurance, with the calculated error defined for each control. SPE was performed using either the CAPILLARYS 2 system (Sebia, France) or agarose gel electrophoresis (HELENA Biosciences, UK). For all adult patients with hypogammaglobulinemia, defined as patients with IgG <6 g/L, immunofixation (HELENA Biosciences, UK) was performed to detect paraproteins.

Secondary causes were investigated in all patients with IgG <6 g/L according to the ICON Document (14). In patients with unexplained hypogammaglobulinemia, IgA and IgM levels were determined. Those with reduced IgA and/or IgM underwent full immunological assessment, including extended B-lymphocyte phenotyping, and were evaluated against the ESID diagnostic criteria for CVID (15).

### 2.3 Statistical analysis:

All statistical analyses were performed using R software (R Foundation, version 4.4.1). Baseline characteristics were summarized as mean ± standard deviation (SD) (for normally distributed continuous variables), as median with interquartile range (IQR) (for non-normally distributed continuous variables), or as percentages (for discontinuous variables). The assumption of normality was assessed using the Shapiro–Wilk test. Depending on data distribution, correlations between IgG and both CG and gammaglobulin levels were evaluated using Pearson’s correlation coefficient (for normally distributed variables) or Spearman’s rank correlation coefficient (for non-normally distributed variables). Linear regression models were applied to examine the association between IgG levels (dependent variable) and CG or gammaglobulin levels (independent variables). The chi-squared test or Fisher’s exact test (for frequencies less than 5) was applied to analyze the association between hypogammaglobulinemia and each clinical category.

Receiver Operating Characteristic (ROC) curve analysis was performed using the *pROC* package (version 1.19.0.1) to determine optimal CG and gammaglobulin cut-off values for detecting hypogammaglobulinemia. Test accuracy was assessed by the area under the curve (AUC), with values >0.8 interpreted as good diagnostic performance (29). The optimal cut-off was defined as the value providing the best balance between sensitivity and specificity (the highest Youden’s Index) (30). Bootstrapping was performed to estimate 95% confidence intervals (CI).

Analyses were conducted for the overall sample, and results were compared between adult and pediatric subgroups. A p-value < 0.05 was considered statistically significant.

## 3 Results

### 3.1 Baseline characteristics:

A total of 980 hospitalized patients were enrolled, comprising 762 adults (≥18 years) and 218 pediatric patients (10 to 17 years). A control group of 20 patients with a confirmed diagnosis of CVID was also included. The overall median age was 47 years (range 10–96), with 46% male participants. The mean age in the adult group was 53 years (range 18–96; 48% male), and 13 years in the pediatric group (range 10–17; 41% male). The control group had a mean age of 27 years, with 50% male participants. Patients were recruited primarily from internal medicine (41%), pneumology (38%), and pediatrics (21%). CG and IgG levels were available for all patients, while gammaglobulins were measured in all adults and in 65 pediatric patients (**Table 1**). Gammaglobulin measurements were performed using capillary SPE in 332 patients, including 39 with hypogammaglobulinemia, and agarose gel SPE in 495 patients, including 55 with hypogammaglobulinemia.

**Table 1:** Baseline characteristics of the overall cohort, adult and pediatric groups, and patients with hypogammaglobulinemia.

Baseline characteristics	Overall cohort	Adult group (≥ 18 years)	Pediatric group (10–17 years)	Cases with IgG <6g/L
Demographic data				
Sample size n (%)	980	762 (78%)	218 (22%)	94 (10%)
Age (years) Median (IQR)	47 (22 – 63)	53 (41 – 65)	13 (11 – 15)	57 (36 – 72)
Sex ratio (M/F)	0.9	0.9	0.8	0.7
Clinical presentation n (%)				
Infections	238 (24%)	215 (28%)	23 (11%)	18 (19%)
Autoimmune diseases	322 (33%)	199 (26%)	123 (56%)	33 (35%)
Inflammatory disorders	307 (31%)	250 (33%)	57 (26%)	23 (24%)
Allergies	31 (3%)	25 (3%)	6 (3%)	2 (2%)
Polyclonal lymphoproliferation	34 (3%)	28 (4%)	6 (3%)	6 (6%)
Malignancy	62 (6%)	60 (8%)	2 (1%)	15 (16%) *
Immunosuppressive therapy	78 (8%)	62 (6%)	16 (7%)	18 (19%) *
Laboratory findings				
IgG (g/L) Median (IQR)	10.8 (8.2 - 13.2)	10.8 (8.1 - 13.4)	10.8 (8.3 - 12.8)	4.7 (3.9 - 5.4)
CG (g/L) Median (IQR)	27.6 (23.6 - 32.1)	27.6 (23.1 - 32.3)	27.7 (24.5 - 31.2)	19.7 (17.5 - 22.2)
Gammaglobulins (g/L) Median (IQR)	11.9 (9.4 - 15.2)	11.9 (9.6 - 15.5)	10.5 (8.8 -13.3)	6.2 (5.1 - 8.3)

IQR, Inter-Quartile Range; SD, Standard Deviation; CG, Calculated Globulin; n, number of patients; M, Male; F, Female.

* Significant association (Chi-squared test, P-value<0.05).

Clinical manifestations included infectious and non-infectious diseases. Autoimmune manifestations were the most frequent (33%), mainly lupus (44 cases, 14%), rheumatoid arthritis (36 cases, 11%), and type 1 diabetes (28 cases, 9%). Inflammatory disorders were the second most common (31%), including liver disease (54 cases, 18%), inflammatory bowel disease (49 cases, 16%), and sarcoidosis (48 cases, 16%). Severe infections were observed in 24% of patients, most often pneumonia (120 cases, 50%). Lymphoma was the most frequent malignancy (24 cases, 39%). In addition, 78 patients (8%) were receiving immunosuppressive therapy (**Table 1**). Associations between hypogammaglobulinemia and clinical manifestations were assessed using the Chi-squared test, showing significant associations only for malignancy and immunosuppressive therapy.

Hypogammaglobulinemia g/L was observed in 94 patients (10%). Secondary causes were investigated according to the ICON Document (14) and were identified in 61 patients (65%), including protein loss (32 patients, 34%), drug-induced hypogammaglobulinemia (18 patients, 19%), and malignancy (11 patients, 12%). Among the remaining 33 patients, primary antibody deficiency was confirmed in 6 cases (6%) according to ESID diagnostic criteria: 3 with CVID, 2 with combined immunodeficiency, and 1 with Good’s syndrome.

### 3.2 Calculated globulin and gammaglobulin correlation with IgG levels:

#### 3.2.1 Calculated globulin

*Overall cohort*

A statistically significant positive correlation between CG and IgG levels was observed in the overall cohort (Spearman’s ρ = 0.65, *p* < 0.05). Linear regression analysis showed that CG variation accounted for 49% of IgG variation.

*Adult group*

**Figure 1:**
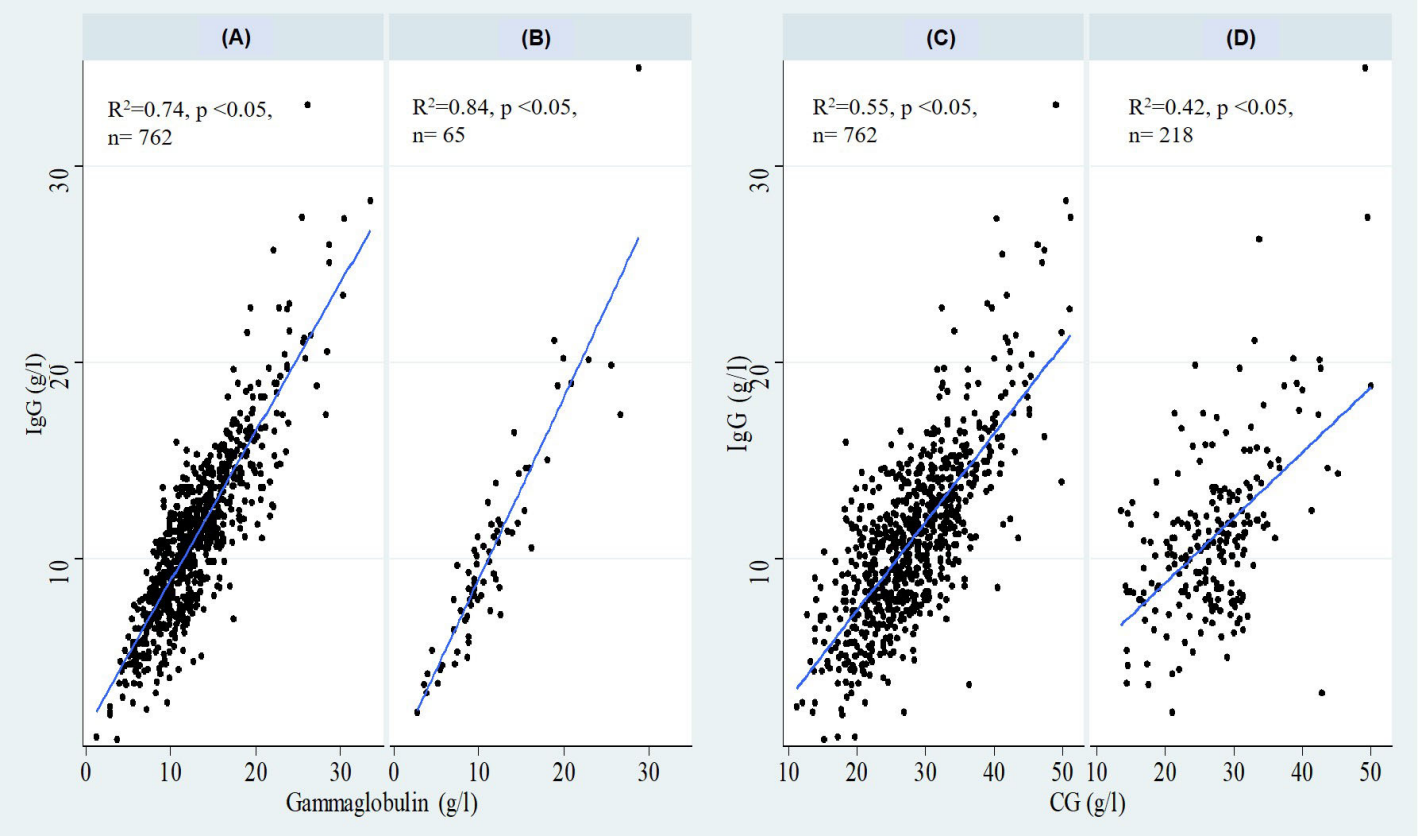
Correlation of IgG with gammaglobulins in the adult group **(A)** and the pediatric group **(B)**, and with CG in the adult group **(C)** and the pediatric group **(D)**. CG, Calculated Globulin; n, number of patients.

A statistically significant positive correlation was also observed between CG and IgG levels in the adult cohort (ρ = 0.70, *p* < 0.05). Linear regression analysis showed that CG variation accounted for 55% of IgG variation (**Figure 1C**).

*Pediatric group*

The correlation was weaker in the pediatric subgroup (ρ = 0.50, *p* < 0.05). Linear regression analysis showed that CG variation accounted for 42% of IgG variation (**Figure 1D**).

#### 3.2.2 Gammaglobulins

*Overall cohort*

**Figure 2:**
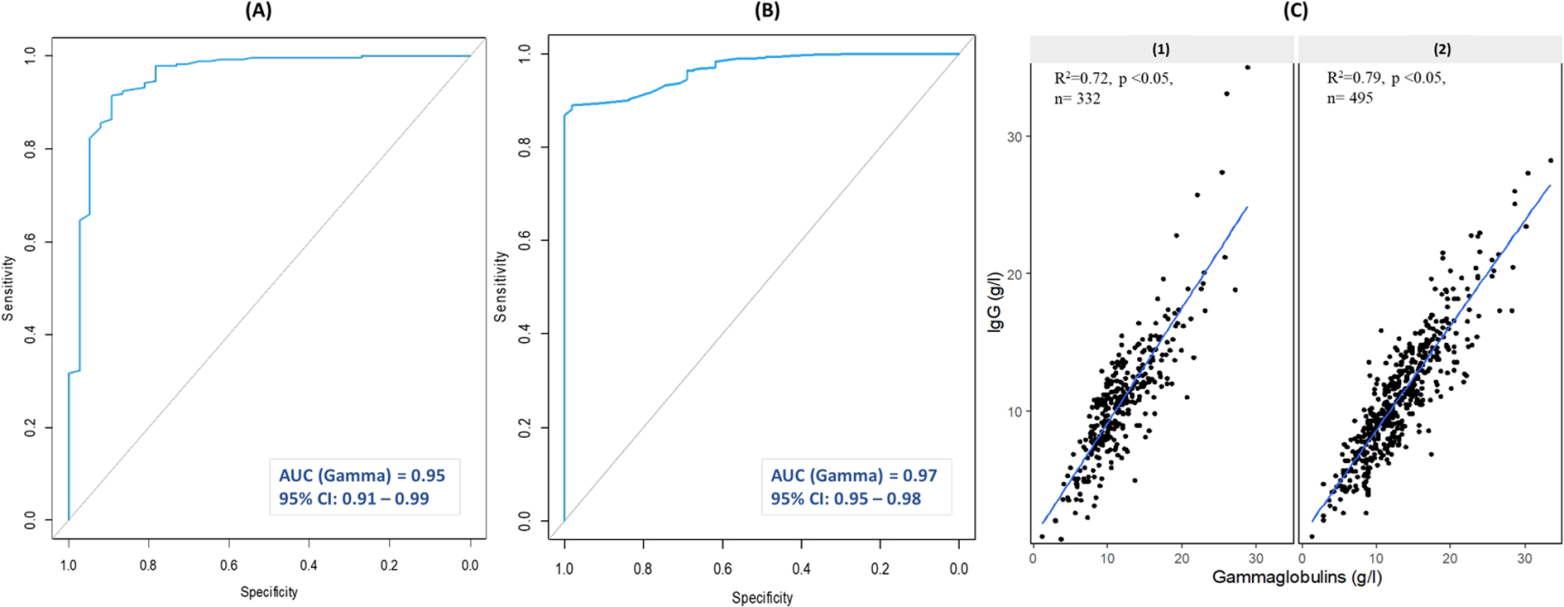
ROC curve analysis of gammaglobulins for detecting IgG <6 g/L using capillary electrophoresis **(A)** and gel electrophoresis **(B)**, and correlation analysis of IgG with gammaglobulins using capillary electrophoresis **(C1)** and gel electrophoresis **(C2)**. AUC, Area Under the Curve; CI, Confidence Interval; n, number of patients.

A strong positive correlation between gammaglobulins and IgG levels was observed in the overall cohort (Spearman’s ρ = 0.84, *p* < 0.05). Linear regression analysis indicated that gammaglobulin variation accounted for 75% of IgG variation. Stratified analysis by electrophoretic technique demonstrated a slightly stronger correlation with agarose gel electrophoresis (**Figure 2C2**) compared with capillary electrophoresis (**Figure 2C1**).

*Adult group*

Strong correlation was also observed in the adult subgroup (ρ = 0.83, *p* < 0.05), and gammaglobulin variation accounted for 74% of IgG variation (**Figure 1A**).

*Pediatric group*

The correlation between gammaglobulins and IgG was stronger compared to the correlation between CG and IgG in the pediatric subgroup (ρ = 0.90, *p* < 0.05). Gammaglobulin variation accounted for 84% of IgG variation (**Figure 1B**).

### 3.3 ROC curve analysis and cut-off determination:

#### 3.3.1 Overall cohort

**Figure 3:**
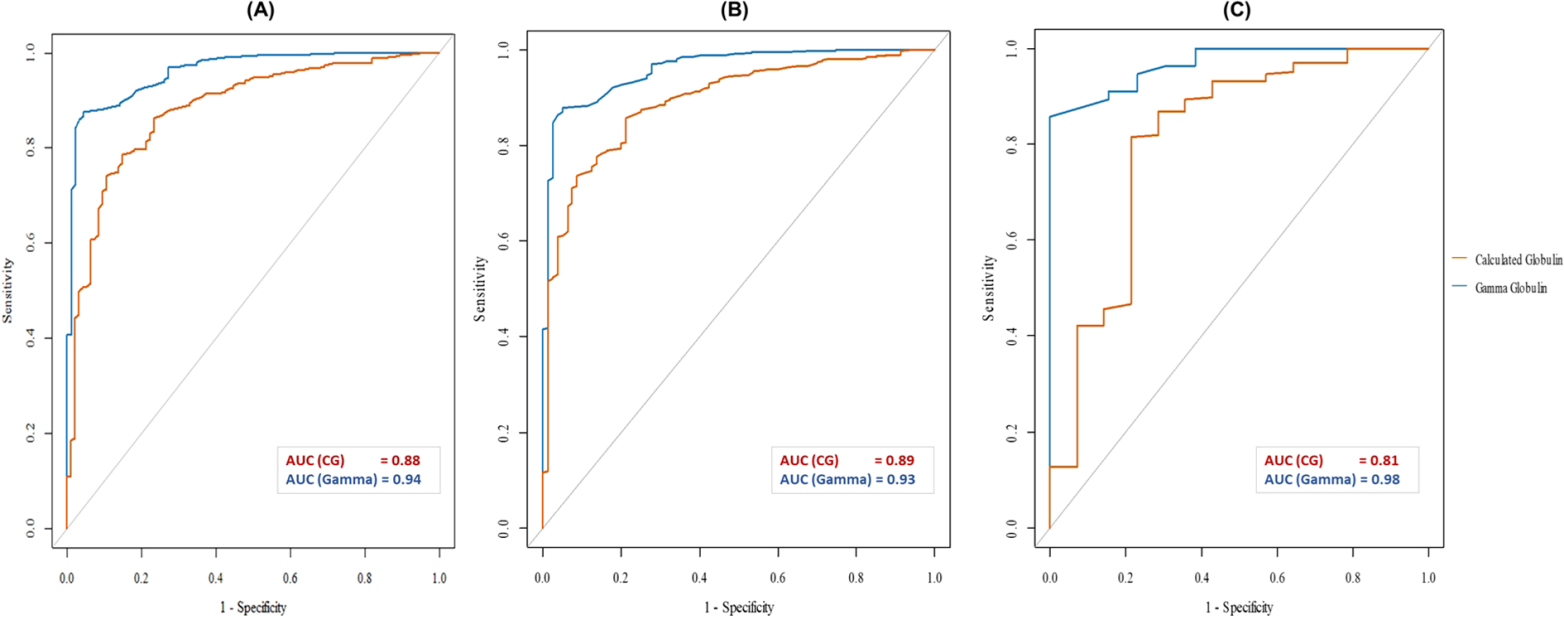
ROC curves of CG and gammaglobulins for detecting IgG <6 g/L in the overall cohort **(A)**, the adult group **(B)**, and the pediatric group **(C)**. CG, Calculated Globulin; Gamma, Gammaglobulins; AUC, Area Under the Curve.

The discriminatory power of both CG and gammaglobulins was good for detecting hypogammaglobulinemia. CG demonstrated an AUC of 0.88 in the overall cohort (95% CI: 0.85 – 0.92). Gammaglobulins showed an excellent discriminatory performance (AUC: 0.94; CI: 0.93 – 0.97) (**Figure 3A**).

A CG cut-off of 22 g/L (95% CI: 21.25 - 24.95 g/L) yielded a sensitivity of 80% and a specificity of 87%, whereas a gammaglobulin cut-off of 8.9 g/L provided a sensitivity of 96% and a specificity of 88% (**Table 2**).

**Table 2:** ROC curve performance and cut-off characteristics for detecting IgG <6 g/L in the overall cohort, the adult, and the pediatric groups.

Patient group	AUC	AUC 95% CI	Cut-off	Cut-off 95% CI	Sensitivity	Specificity	Youden’s Index	Cases below cut-off n (%)
Calculated Globulin								
Overall cohort n=980	0.88	0.85 – 0.92	22.00	21.25 – 24.95	0.80	0.87	0.67	205 (21%)
Adult group n=762	0.89	0.86- 0.92	22.25	21.95 – 25.45	0.79	0.86	0.65	161 (21%)
Pediatric group n=218	0.81	0.69 – 0.99	24.15	21.05 – 24.25	0.81	0.79	0.60	40 (18%)
Gammaglobulins								
Overall cohort n=980	0.94	0.93 – 0.97	8.9	8.80 – 9.25	0.96	0.88	0.87	180 (18%)
Adult group n=762	0.93	0.91 – 0.98	8.9	8.80 – 9.25	0.95	0.89	0.84	150 (20%)
Pediatric group n=65	0.98	0.95 – 1.00	8.9	6.34 - 9.13	1.00	0.84	0.84	22 (34%)

AUC, Area Under the Curve; CI, Confidence Interval; n, number of patients.

Slight variations in cut-off values were observed between techniques: gel SPE, 8.9 g/L (sensitivity 98%, specificity 89%); CAPILLARYS 2 SPE, 8.45 g/L (sensitivity 89%, specificity 91%). Stratified ROC curves are demonstrated in **Figures 2A, B**, respectively.

#### 3.3.2 Adult group

CG demonstrated an AUC of 0.89 in the adult group (CI: 0.86 – 0.92). Gammaglobulins also demonstrated excellent discriminatory performance (AUC: 0.93; 95% CI: 0.91–0.98) (**Figure 3B**).

A CG cut-off of 22.25 g/L (95% CI: 21.95 - 25.45 g/L) yielded a sensitivity of 79% and a specificity of 86%, whereas a gammaglobulin cut-off of 8.9 g/L provided a sensitivity of 95% and a specificity of 89% (**Table 2**).

#### 3.3.3 Pediatric group

CG demonstrated an AUC of 0.81 in the pediatric group (95% CI: 0.69 – 0.99). In contrast, gammaglobulins showed excellent discriminatory performance (AUC: 0.98; 95% CI: 0.95 – 1.00) (**Figure 3C**).

A CG cut-off of 24.15 g/L (95% CI: 21.05 - 24.25 g/L) yielded a sensitivity of 81% and a specificity of 79%, whereas a gammaglobulin cut-off of 8.9 g/L provided a sensitivity of 100% and a specificity of 84% (**Table 2**).

### 3.4 Control group:

A cohort of 20 patients with CVID, diagnosed according to ESID criteria, was used as a control group to test the performance of the CG cut-off. All patients had IgG levels <6 g/L (mean ± SD: 3.7 ± 2.1 g/L). The mean CG level was 22.0 ± 4.4 g/L, and 13 of the 20 patients (65%) had a CG level below 22 g/L. IgG and CG levels were measured before initiating Ig replacement therapy.

## 4 Discussion

Although predominantly antibody deficiencies are considered the most frequent forms of IEI, with CVID being the most common symptomatic PAD, early diagnosis remains a major challenge, particularly in countries with limited resources and experience in managing IEI. Low-cost screening tools are therefore essential for patients presenting with key features such as recurrent infections, autoimmunity, inflammation, polyclonal lymphoproliferation, or malignancy (7, 31, 32). CG has been investigated in several studies and shown to meet the criteria for effective screening tests for hypogammaglobulinemia (17, 33). To our knowledge, this study is the first conducted in Africa and the MENA region to validate and establish a CG cut-off as a screening marker for antibody deficiency, in a cohort of hospitalized adult and adolescent patients presenting with recurrent infections and/or immune dysregulation.

The demographic analysis revealed that most patients presented with autoimmune, inflammatory, or infectious manifestations. The notably high prevalence of immune dysregulation in hospitalized adolescents emphasizes the importance of screening for IEI in this age group. Interestingly, although autoimmune diseases were the most frequent diagnosis among patients with hypogammaglobulinemia, no significant association was observed. In contrast, strong associations were found with malignancy and immunosuppressive therapy, highlighting the importance of considering secondary causes when evaluating hypogammaglobulinemia.

The analysis of the underlying causes of hypogammaglobulinemia in the 94 affected patients revealed that secondary causes, as defined by ICON criteria (14), accounted for 65% of cases, whereas primary causes represented only 6%. This finding is consistent with the literature, which reports that secondary hypogammaglobulinemia is approximately 30 times more common than primary forms (34). Recent studies have emphasized the importance of monitoring secondary hypogammaglobulinemia and, in selected cases, initiating Ig replacement therapy to prevent complications (34, 35). In this context, simple screening methods may play an important role in tracking disease progression and can be particularly useful in settings where direct measurement of Ig levels is not available.

A strong positive correlation was observed between IgG and both CG and gammaglobulin levels in the overall cohort, consistent with findings from nearly all comparable studies (17, 19–24, 26, 27, 36). Notably, comparative analysis between the adult and pediatric groups revealed that the correlation between CG and IgG was weaker in the pediatric subgroup, whereas gammaglobulins maintained a stronger association with IgG levels. These differences may be attributed to age-related variations in protein production, which influence CG values more substantially than gammaglobulin levels, the latter primarily reflecting serum Ig concentrations.

The AUC is the most widely used statistical parameter for validating ROC curves. According to commonly applied classifications, test performance is considered excellent when AUC >0.9, considerable when 0.8 < AUC < 0.9, fair when 0.7 < AUC < 0.8, and poor when 0.6 < AUC < 0.7 (29). In our study, ROC curve analysis yielded satisfactory AUC values for both CG and gammaglobulins across all age groups (AUC >0.8). The 95% CI were consistently narrow, apart from CG in the pediatric group, where the interval was considerably wider (95% CI: 0.69–0.99) (**Table 2**). This may reflect the weaker correlation between CG and IgG in this subgroup, as well as the smaller number of hypogammaglobulinemia cases among the pediatric group (14 cases) compared with adults (80 cases). Although the AUC values support the validity of the ROC curve analysis, it should be acknowledged that the data distribution was skewed toward higher CG values, despite adequate representation of low CG levels (205 samples below 22 g/L (**Table 2**)). Consequently, these results should not be interpreted as a fully generalizable model.

The optimal CG cut-off for detecting IgG <6 g/L in the overall cohort was 22 g/L. Differences were observed between age groups, with cut-offs of 22.25 g/L in adults and 24.15 g/L in the pediatric group (**Table 2**). Reported cut-offs using the same laboratory methodology (biuret method for TP and bromocresol green for albumin) have varied across studies (**Table 3**). Jolles et al. proposed 18 g/L (sensitivity 66%, specificity 78% for IgG <5 g/L), Pecoraro et al., 19 g/L (sensitivity 70%, specificity 75%), Yegit et al., 20 g/L (sensitivity 83.8%, specificity 74.9%), and Piza et al., 23.1 g/L in adolescents aged 10–18 years (sensitivity 76.3%, specificity 100%) (17, 20, 23, 27). Our findings are consistent with the Brazilian study conducted in children under 18 years of age, as a difference of approximately 1 g/L was observed between the two cut-off values (27). The difference observed in the adult cut-off value may be attributable to differences in recruitment methodology. The original study validating CG as a screening tool for antibody deficiency recruited 50 samples at each CG level, ranging from 15 to 22 g/L, using analytical methods comparable to those applied in our study (17). This strategy ensured a normal data distribution and adequate representation across CG thresholds. Such an approach could not be applied in our cohort, as the study was conducted prospectively over a one-year period. Moreover, a retrospective design was not feasible since CG is not routinely measured in Algeria, and one of the objectives of this study was to introduce CG as a screening parameter. Finally, 95% CI were determined for the cut-off values (**Table 2**), and Ig measurement is strongly recommended when a CG value falls within the corresponding 95% CI.

**Table 3:** Comparison of study results with major similar studies worldwide.

Study	Patients	IgG Threshold	CG Cut-off (g/L) (sen, spe)	Gammaglobulins cut-off (g/L) (sen, spe)	Ref.
Current study	762 adults 218 Children between 10 and 17 years old	<6 g/L	<22.25 (79%, 86%) <24.15 (81%, 79%)	<8.9 (95%, 89%) <8.9 (100%, 84%)	/
Jolles et al., 2014, Wales	400 adults (50 samples per CG range, 15–22 g/L).	<5 g/L	<18 (66%, 78%)	ND	(17)
Pecoraro et al., 2018, Italy	200 adults (25 samples per CG range, 15–22 g/L).	<6 g/L	<19 (70%, 75%)	ND	(20)
Yegit et al, 2023, Turkey	500 adults (50 samples per CG range, 15–25 g/L).	<6 g/L	<20 (83.8%, 74.9%)	<7 (89%, 89.4%)	(23)
De Toledo Piza et al., 2024, Brazil	309 children between 10 and 18 years old	<6 g/L	<23.10 (76.3%, 100%)	ND	(27)

CG, Calculated Globulin; Ref, References; Sen, Sensitivity; Spe, Specificity; ND, Not Determined.

A CG cut-off of 22 g/L identified 65% of CVID patients (13/20). Although all patients in this group had IgG levels <6 g/L, seven presented with higher CG values. These findings confirm that CG may be less reliable in patients with active infection or acute inflammation, in whom elevated α- and/or β-globulin fractions can mask low IgG concentrations. In addition, albumin is a negative acute-phase protein, which further contributes to this limitation, as decreased albumin levels during inflammatory states may increase CG values. Consequently, patients with CVID may be overlooked during episodes of infection or during active inflammatory or autoimmune phases. Therefore, direct Ig measurement remains essential in patients in whom antibody deficiency is strongly suspected.

Our study also evaluated SPE as a reliable tool for detecting hypogammaglobulinemia. Unlike CG, which reflects all serum proteins except albumin, SPE provides a semi-quantitative assessment of five or six protein fractions. Although gammaglobulin results can be presented either as a percentage or as a concentration based on TP, the absolute concentration values were preferred to facilitate comparison between the three parameters (gammaglobulins, CG, and IgG), since albumin, used in CG determination, is calculated separately. Because IgG is a major component of the gamma-fraction, measurement of gammaglobulins offers a clearer indication of IgG levels. In our cohort, we demonstrated a stronger correlation between IgG and gammaglobulin levels compared with CG, and the gammaglobulin cut-off yielded superior sensitivity and specificity. These findings are consistent with most studies that have investigated both indices (21, 23, 24). Interestingly, our gammaglobulin cut-off was higher than those reported in most previous studies and by manufacturers. This may reflect differences in the analytical techniques used, as well as the heterogeneity of our cohort, which included patients with conditions associated with increased production of other immunoglobulin isotypes (e.g., liver disease, severe infections). We also observed slight differences in cut-off values between the two SPE techniques applied: 8.45 g/L for CAPILLARYS 2 and 8.9 g/L for HELENA gel SPE, emphasizing the importance of establishing local cut-offs tailored to the SPE technique employed.

Although our study revealed interesting findings, it has certain limitations. The heterogeneity of the study population allowed us to propose a CG cut-off applicable to hospitalized patients with diverse clinical presentations; however, further studies focusing on specific clinical subgroups may provide more refined and clinically relevant CG cut-offs. In addition, we evaluated the screening performance of CG using serum IgG concentration as the reference standard, meaning that deficiencies in other Ig isotypes could not be detected, such as selective IgA deficiency. Another limitation of this study is that data skewness toward higher CG values may compromise the stability and generalizability of the derived cut-off. Future studies should therefore include larger cohorts with a more balanced and normally distributed sample, especially with greater representation of CG values below 22 g/L. Furthermore, two different electrophoretic methods were used to determine gammaglobulin concentrations without formal demonstration of their interchangeability. Although the observed differences in cut-off values between the two techniques were minimal, this methodological variability remains a limitation. Finally, multicenter studies incorporating larger populations and additional laboratory techniques are needed to confirm these findings and support their broader generalization.

In conclusion, our study confirms the utility of CG and SPE as screening tools for antibody deficiency in hospitalized patients with heterogeneous clinical manifestations. CG offers high sensitivity at a lower cost, whereas SPE provides additional diagnostic information by assessing all protein fractions, including the gamma fraction, and by detecting paraproteins. Although both tests demonstrated satisfactory sensitivity and specificity, certain cases may be underestimated or overestimated. Therefore, thorough clinical evaluation and, whenever possible, direct Ig measurement remain essential.

## Data Availability

The raw data supporting the conclusions of this article will be made available by the authors, without undue reservation.
